# Suicide

**Published:** 1858-08

**Authors:** 


### SUICIDE.

About as many people kill themselves in England as in France, according to the population—three or four thousand a year. In a previous number, it was stated, that crime most abounded in summer-time in England; and the same is the case as to suicide; it is oftenest resorted to, not during the fogs of November and the piercing cold of mid-winter; it is in the merry month of May, in flowering June, and in the glad sunshine of July. Largely over three hundred court death in a summer month, while chill December does not give two hundred. It is one of the rarest of all occurrences to hear of a man's drowning himself in midwinter; the very idea of being frozen to an icicle is repulsive!

It is a matter of considerable practical importance to ascertain the cause of the increase of crime and suicide at a season of the year when all Nature is so full of flowers, and sunshine, and gladness, for we would naturally suppose these were circumstances calculated to increase our love of life.

We believe that the question is fully and philosophically answered in one word: "Idleness."

> "For Satan finds some mischief still
> For idle hands to do,"

is as true now as when first sung by the immortal Isaac Watts.

Men who have half a dozen irons in the fire, are not the ones to go crazy. It is the man of voluntary or compelled leisure who mopes, and pines, and thinks himself into the madhouse, or the grave. Motion is all Nature's law. Action is man's salvation, physical and mental. And yet, nine out of ten are wistfully looking forward to the coveted hour, when they shall have leisure to do nothing, or something, only if they feel like it—the very Siren that has lured to death many a "successful" man.

He only is truly wise who lays himself out to work till life's latest hour, and that is the man who will live the longest, and will live to most purpose.

As to the body, the summer heats relax, invite to physical inactivity and ease; locomotion is an effort; the mind itself participates in the inertia of the body, and both stagnate together. On the contrary, the sparkling frosts of winter rouse up our activities, the pulses bound with the fire of life, and we are ready, at a moment's notice, to do or dare anything; we can scarce keep the body still; motion is a luxury, while in summertime it was a drag. The great practical lesson is, in proportion as you would avoid crime and madness, aim to be *fully* employed, whether in summer or winter, in doing something which combines, in its highest extent, the useful and the good.

